# Oral Soft Tissue Metastasis from Breast Cancer as the Only Primary Source: Systematic Review

**DOI:** 10.1055/s-0044-1779674

**Published:** 2024-02-27

**Authors:** Nausheen Aga, Ruchira Shreevats, Sonia Gupta, Harman Sandhu, Muna E.M. Hassan, Harnisha V. Prajapati

**Affiliations:** 1 School of Dentistry, University of Dundee, Dundee, Scotland, United Kingdom; 2 Department of Orthodontics, Primadent Dental Centre, Bangalore, Karnataka, India; 3 Department of Oral Pathology and Microbiology and Forensic Odontology, Yamuna Institute of Dental Sciences & Research, Gadholi, Yamunanagar, Haryana, India; 4 General Dentistry, Building Smiles Dental Clinic, Mohali, Punjab, India; 5 Department of Preventive and Restorative Dentistry, University of Sharjah, United Arab Emirates; 6 General Dentistry, Bhavya Dental Clinic and Implant Centre, Palanpur, Gujarat, India

**Keywords:** breast cancer, metastasis, oral soft tissues

## Abstract

**Background** Breast cancer is one of the most lethal neoplasms causing death. Oral cavity is the rare site of distant metastasis from breast cancer. Very little research has been conducted to date to analyze breast cancer as the sole primary source of metastasis to the oral soft tissues. The goal of this study was to examine the published cases of oral soft tissue metastasis from breast cancer as the only primary source to date.

**Methods** An electronic search of the published literature was performed without publication year limitation in PubMed/Medline, Scopus, Google Scholar, Web of Science, Science Direct, Embase, and Research Gate databases, using mesh keywords like (“Breast cancer”, OR “Breast carcinoma”) AND (“Metastasis” OR “Metastases”), And (“Oral soft tissues” OR “Tongue” OR “Palate” OR “Tonsil” OR “Buccal mucosa” OR “Floor of mouth” OR “Vestibule” OR “Salivary glands”). We also searched all related journals manually. The reference list of all articles was also checked.

**Results** Our research revealed 88 relevant papers (September 1967–September 2023) with 96 patients in total. The most predominant oral soft tissues involved were salivary glands followed by the gingiva, tonsils, tongue, and buccal mucosa. A total of 23% of patients died with an average survival time of 1 to 15 months.

**Conclusions** Oral soft tissue metastasis from breast cancer is a rare event and has a bad prognosis. More cases need to be published to raise awareness of these lesions.

## Introduction

Breast cancer (BC) is one of the most lethal neoplasms causing death. Worldwide, approximately 2.3 million new cases of BC and 684,996 deaths due to this malignancy were recorded in 2022 according to GLOBACON databases 1 and metastasis is the prime cause of death. The rate of metastasis even in uncommon sites is on the rise. On the other side, it has been observed that the overall survival of BC patients has been prolonged owing to the more effective therapy and the development of new imaging techniques and early detection. The most common organs involved in distant metastasis of BC are bones, lungs, liver, and brain. 2 The oral cavity is the rarest site of metastasis, and it can involve both osseous and soft tissues. Lung cancer is the most common cancer metastasizing to the oral soft tissues (OST), whereas BC is the most common source of metastasis to the jawbones (JB). 3 The prognosis of metastatic lesions in the oral cavity is unfavorable because of their late detection owing to the resemblance of benign growths. Literature has reported several studies analyzing metastatic tumors in the oral region. 3 4 But very little research has been conducted to date to analyze BC as the sole primary source of metastasis to the OST. The goal of this study was to examine the published cases of oral soft tissue metastasis (OSTM) from BC as the only primary source to date.

## Materials and Methods

The current research was performed following the guidelines of Preferred Reporting Items for Systematic Reviews and Meta-Analyses. Owing to nature of the current review, any ethical approval was not required.

### Focused Question

To conduct the study, CoCoPop (context, condition, population) framework, designed by Joanna Briggs Institute, was used focusing on the research question “How many cases of BC metastasizing to OST have been documented in the literature to date, and what is the prognosis of these metastatic lesions”?

- Pop (population): patients with BC.
- Co (condition): salivary gland metastasis.
- Co (context): characteristics of these patients.

### Search Strategy for Identification of Studies

**Fig. 1:**
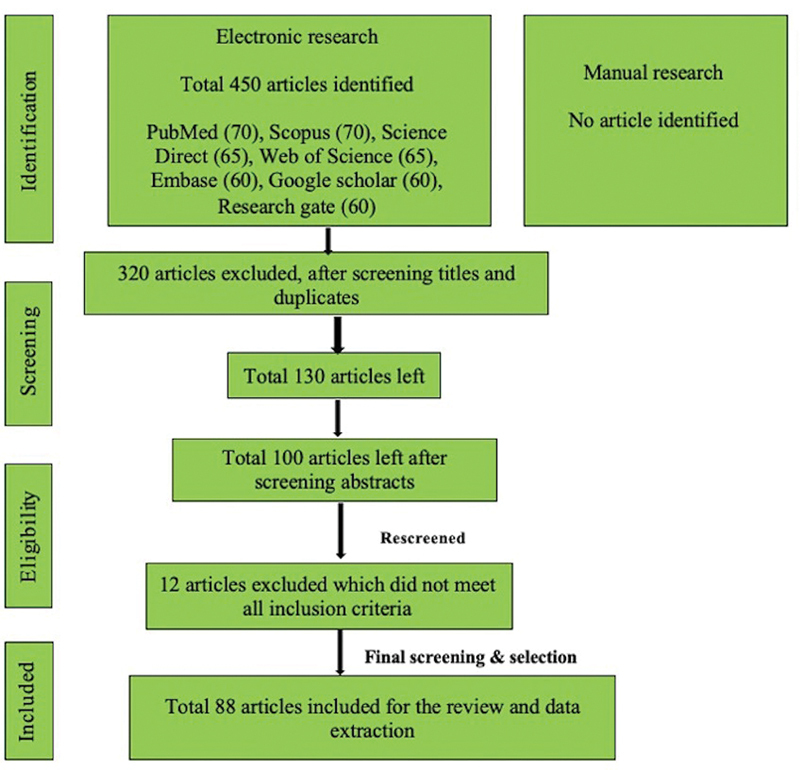
PRISMA flowchart showing search strategy. PRISMA, Preferred Reporting Items for Systematic Reviews and Meta-Analyses.

An electronic search of the published literature was performed without publication year limitation in PubMed/Medline, Scopus, Google Scholar, Web of Science, Science direct, Embase, and Research Gate databases, using mesh keywords like (“Breast cancer”, OR “Breast carcinoma”) AND (“Metastasis” OR “Metastases”), And (“Oral soft tissues” OR “Tongue” OR “Palate” OR “Tonsil” OR “Buccal mucosa” OR “Floor of mouth” OR “Vestibule” OR “Salivary glands”). We also searched all related journals manually. The reference list of all articles was also checked ( Fig. 1 ).

### Screening of Studies

The current review involved three steps of screening the studies. In the first step, titles were reviewed by two authors (N.A., R.V.) independently and duplicates were removed. Then the other two authors (S.G., H.S.) reviewed the selected abstracts of all the reports independently. The reviewers were calibrated on the basis of their assessment of their titles and abstracts of the first 50 references retrieved. The kappa value of agreement between reviewers was 0.84. If the title/abstracts met the eligibility rule, they were included in the study. In the final stage, the text of selected studies was screened by remaining two authors (M.E.M.H., H.V.P.) separately. The full report was collected, discussed, and resolved for cases among all authors that appeared to fit the inclusion criteria or for which evidence was insufficient to make a clear determination.

### Inclusion Criteria

- Confirmed cases of OSTM from BC as the sole primary source. The papers included were from September 1967 to September 2023.
- Type of studies: case reports, case series, retrospective analysis, and original research.
- Cases were selected beyond the restriction of limitations on parameters such as age, gender, ethnicity socioeconomic status, etc.
- Articles published in any language were included.

### Exclusion Criteria

- Cases with no definite diagnosis of OSTM from BC as the sole primary source.
- Publications reporting the OSTM from any site other than breast.
- Cases with BC metastasis to JB and paranasal sinuses were not included.
- Studies that didn't provide individual patient data were excluded.
- Review articles, editorials, conference abstracts, hypothesis papers, web news, media reports, and animal studies.

### Outcome Measures

Primary outcome measures: to evaluate the number of cases of OSTM from BC as the sole primary source reported in the literature and to determine their prognosis.
Secondary outcome measures: to evaluate other factors such as worldwide distribution of cases of OSTM from BC, patient's demographic details, the predominant site of OSTM, clinical features of these metastatic lesions, most prevalent type of metastatic BC, immunoprofile, type of therapies used, and the prognosis of these patients.

### Risk of Bias Assessment

Most of the studies included in this review were case reports and case series. The risk of bias was appraised following CARE and Strengthening the Reporting of Observational Studies in Epidemiology checklists. 5 6 In several papers, there was missing information regarding many parameters used for data extraction. We tried reaching the authors of those cases to clarify this bias; however, we were unable to recover the missing information.

### Data Extraction and Analysis

After study selection, screening and a thorough examination, the data were extracted. The information gathered was cross-checked and tabulated into three tables ( Tables 1 2 3 4 ). In case of missing data, 6 weeks' time was given to gather the information. If the information was still missing, we then indicated the missing data as “not available” in the text and in the tables. The results were expressed in descriptive statistics. The overall survival rate was calculated by survival analysis with Kaplan–Meier curves.

**Table 1:** Details of publications reporting cases of breast cancer metastasizing to oral soft tissues (September 1967–September 2023) 7 8 9 10 11 12 13 14 15 16 17 18 19 20 21 22 23 24 25 26 27 28 29 30 31 32 33 34 35 36 37 38 39 40 41 42 43 44 45 46 47 48 49 50 51 52 53 54 55 56 57 58 59 60 61 62 63 64 65 66 67 68 69 70 71 72 73 74 75 76 77 78 79 80 81 82 83 84 85 86 87 88 89 90 91 92 93 94

S. no.	Authors	Year	Country	Type of study	Total no. of patients
1.	Meher-Homji et al	1967	India	CR	1
2.	Perlmutter et al	1974	Israel	CR	1
3.	Solomon et al	1975	United States	CR	1
4.	Barton et al	1980	Durham	CR	1
5.	Meyers and Olshok	1981	United States	CR	1
6.	Wiesel et al	1982	Israel	CR	3
7.	Eckardt and Nommels	1986	Germany	CR	1
8.	Epstein et al	1987	Canada	CR	1
9.	Rosti et al	1987	UK	CR	1
10.	Cooney et al	1988	United States	CR	1
11.	Bissett et al	1989	UK	CR	2
12.	Needleman and Salah	1992	Australia	CR	1
13.	Win et al	1992	Japan	CR	1
14.	Calvo Boizas et al	1995	Spain	CR	1
15.	Vessecchia et al	1995	Italy	CR	1
16.	Bochnia et al	1997	Poland	CR	1
17.	Kollias and Gill	1997	UK	OR	3
18.	Rajesh et al	1998	India	CR	1
19.	Tueche et al	1999	Belgium	CR	1
20.	Joycee et al	2000	Ireland	CR	1
21.	Nicol and Iskandar	2000	United States	CR	1
22.	Cain et al	2001	UK	OR	1
23.	Scipio et al	2001	West Indies	CR	1
24.	Szymanski et al	2002	Poland	CR	2
25.	Zhang and Gu	2003	United States	CR	1
26.	Adelson et al	2005	United States	CR	1
27.	Chatterjee et al	2006	UK	CR	1
28.	Malhotra et al	2006	India	CR	1
29.	Masmoudi et al	2006	Tunisia	CR	1
30.	Nuyens et al	2006	UK	RA	2
31.	Perez-Fidalgo et al	2007	Spain	CR	1
32.	Neelakantan et al	2008	UK	CR	1
33.	Billan et al	2009	Israel	CR	1
34.	Dangore-Khasbage et al	2009	India	CR	1
35.	Laforga and Gasent	2009	Spain	CR	1
36.	Shah and Mehta	2009	India	CR	1
37.	Ramesh et al	2010	India	CR	1
38.	Bar et al	2011	Israel	CR	1
39.	Cihan et al	2011	Turkey	CR	1
40.	Erra and Costamagna	2011	Italy	CR	1
41.	Sellinger et al	2011	Germany	CR	1
42.	Al-Benna and Tzakas	2012	UK	CR	1
43.	Kechagias et al	2012	Greece	CR	1
44.	Maruzzo et al	2012	Italy	CR	1
45.	Jain et al	2013	India	CR	1
46.	Addeo et al	2014	Italy	CR	1
47.	Alath et al	2014	Kuwait	RA	1
48.	Sano et al	2014	Japan	CR	1
49.	Vivas et al	2014	Brazil	CS	1
50.	Akcan et al	2015	Turkey	CR	1
51.	Dievel et al	2015	Belgium	CR	1
52.	Duncan et al	2015	UK	CR	1
53.	Murhekar et al	2015	India	CR	1
54.	Khuranna et al	2016	India	CR	1
55.	Kmeid et al	2016	Lebanon	CR	1
56.	Srinivasan	2016	United States	CR	1
57.	El M'rabet et al	2017	Africa	CR	1
58.	Franzan et al	2017	Germany	RA	1
59.	Rewat et al	2017	UK	CR	1
60.	Sera et al	2017	Japan	CR	1
61.	Yoshiba et al	2017	Japan	CR	1
62.	Bohli et al	2018	Tunisia	CR	1
63.	Cao et al	2018	China	CR	1
64.	Aggarwal et al	2019	India	CR	1
65.	Assarian et al	2019	India	CR	1
66.	Cengiz et al	2019	Turkey	CR	2
67.	de Almeida Freire et al	2019	Brazil	CR	1
68.	Jakharia-Shah et al	2019	UK	CR	1
69.	Thakur et al	2019	India	CR	1
70.	Abdalla et al	2020	UK	CR	1
71.	Andinata et al	2020	Indonesia	CR	1
72.	Dhia et al	2020	Tunisia	CR	1
73.	Medayil	2020	India	CR	1
74.	Ndiaye et al	2020	Senegal	CR	1
75.	Nwabuoku et al	2020	Nigeria	CR	1
76.	Razmara et al	2020	Iran	CR	1
77.	Swain et al	2020	India	CR	1
78.	Jung et al	2021	South Korea	CR	1
79.	Murgia et al	2021	Italy	CR	1
80.	Nikolova et al	2021	Cyprus	CR	1
81.	Waruola et al	2021	Nigeria	CR	1
82.	Menezes et al	2022	Brazil	CR	1
83.	Miyazaki et al	2022	Japan	CR	1
84.	Sadasivan et al	2022	India	CR	1
85.	Almeida et al	2023	Portugal	CR	1
86.	Gholami et al	2023	Iran	CR	1
87.	Mansikka et al	2023	Finland	CR	1
88.	Peron et al	2023	Brazil	CR	1

Abbreviations: CR, case report; CS, case series; OR, original research; RA, retrospective analysis.

**Table 2:** Clinical data of patients with breast cancer metastasizing to oral soft tissues (September 1967–September 2023)

Patient no.	Sex	Age (y)	PHOBC	OST involved (site)	Clinical features	OST as initial site of metastasis	Time of diagnosis of metastasis	Any other site of metastasis	Final diagnosis of metastatic BC	Side of BC
1.	NA	NA	NA	T (Ant)	NA	NA	NA	NA	NA	NA
2.	NA	NA	NA	G (SNA)	Pedunculated mass	NA	NA	NA	NA	NA
3.	F	NA	NA	SMG (NA)	Swelling	NA	NA	NA	NA	NA
4.	F	33	Y	To (L)	NA	N	11 mo	NA	IDC	R
5.	NA	NA	Y	SMG (NA)	Swelling	N	19 mo	NA	NA	NA
6.	F	62	N	P (L)	Swelling	Y	–	NA	ILC	L
7.	F	61	N	P (L)	Swelling	Y	–	NA	IDC	L
8.	F	74	N	P (L)	FNP	Y	–	NA	NA	L
9.	F	68	Y	SMG (NA)	Swelling	N	4 y	NA	NA	NA
10.	F	38	Y	G (Max, R, Ant)	Ulcerative mass	NA	NA	N	IDC	R
11.	NA	NA	Y	G (SNA)	Swelling	N	NA	NA	AS	NA
12.	NA	NA	Y	NA	NA	N	NA	NA	CP	NA
13.	F	41	Y	P (L)	FNP	N	10 y	NA	IDC	R
14.	F	65	Y	P (L)	Swelling	N	NA	NA	IDC	R
15.	NA	NA	N	G (SNA)	Epulis-like growth	Y	NA	NA	CP	NA
16.	NA	NA	NA	G (Max)	Ulcerative mass	NA	NA	NA	AS	NA
17.	F	57	NA	P (L)	Pain	NA	NA	NA	IDC	R
18.	NA	NA	Y	SMG (NA)	Soft painless swelling	N	24 y	NA	IDC	NA
19.	F	42	N	P (BL)	Painless, elastic swelling	Y	–	N	IDC	L
20.	F	52	N	P (R)	Mass	Y	–	N	IDC	R
21.	F	57	N	P (L)	Mass	Y	–	N	IDC	L
22.	NA	NA	N	P (R)	Mass	Y	–	NA	NA	NA
23.	F	40	Y	G (Max, L, Ant)	Swelling	N	NA	NA	IDC	NA
24.	F	71	Y	To (L)	Swelling	N	24 y	N	IDC	NA
25.	NA	71	NA	P (R)	FNP	NA	–	NA	IDC	R
26.	F	54	Y	FOM	Tumor growth	N	12 y	N	ILC	L
27.	NA	NA	NA	SMG (L)	Painless mass	NA	NA	NA	NA	NA
28.	NA	NA	Y	G, (Mand, L, Post)	Pedunculated mass	N	1 y	N	IDC	NA
29.	F	66	Y	P (L)	FNP	N	15 y	NA	IDC	R
30.	F	58	N	P (R)	Swelling	Y	–	NA	IDC	R
31.	F	40	Y	P (R)	Mass	N	NA	NA	MPT	L
32.	F	48	Y	Vestibule (R)	Painful mass	N	24 y	LN	IDC	L
33.	F	44	Y	Multiple sites	Hypoesthesia, tender lymph nodes	N	7 y	N	IDC	NA
34.	F	33	Y	G (Max)	Exophytic growth	N	2 y	Lung	IDC	R
35.	F	44	Y	G (BL)	Exophytic, erythematous mass	N	2 y	Skin	PS	NA
36.	F	NA	NA	P (NA)	Swelling	NA	–	NA	IDC	NA
37.	F	NA	NA	P (NA)	Swelling	NA	–	NA	IDC	NA
38.	F	61	Y	P (L)	Swelling	N	5 y	NA	IDC	L
39.	F	52	Y	P (L)	Swelling	N	2 y	NA	IDC	L
40.	F	24	Y	T (L, border)	Ulcerative lesion	N	10 mo	Lung	IDC	R
41.	F	53	Y	T (R Base)	Lump	N	3 mo	N	TN	L
42.	F	42	Y	P (L)	Swelling	N	1 y	N	IDC	R
43.	F	25	Y	G (Max, Ant)	Swelling	N	1 y	N	IDC	L
44.	F	53	N	P (R)	Swelling	Y	–	N	UD	R
45.	F	54	Y	To (L)	Necrotic mass	N	1 y	MM	HS	R
46.	F	65	Y	P (L)	Swelling	N	11 mo	NA	ILC	L
47.	F	50	Y	SMG (R)	NA	N	9 y	NA	IDC	R
48.	F	74	Y	P (R)	FNP	N	4 y	Bone	ILC	L
49.	F	52	Y	Submental region	Swelling	N	3 y	MM	IDC	NA
50.	F	52	NA	BM (L)	Nodule	NA	NA	NA	IDC	NA
51.	F	74	Y	To (R)	Tender mass	N	23 y	Cervical LN	IDC	L
52.	F	30	Y	BM (R)	Swelling	N	1 y	Axilla	IDC	L
53.	F	81	Y	T (L)	Nodule	N	6 y	Axilla	IDC	L
54.	F	43	N	P (L)	NA	Y	–	Bone, Liver	IDC	L
55.	F	54	Y	To (BL)	Pain	N	3 y	N	MPT	R
56.	F	53	Y	RM (L)	Swelling	N	20 d	MM	SM	L
57.	F	61	N	P (R)	Swelling	Y	–	MM	IDC	R
58.	F	89	Y	T (R)	Swelling	N	4 y	Lung, liver	IDC	R
59.	F	76	Y	P (R)	NA	N	25 y	N	IDC	R
60.	F	60	Y	P (R)	Hard swelling	N	7 y	N	IDC, mucinous	R
61.	F	61	Y	P (R)	Swelling	N	1 y	N	IDC	R
62.	F	65	Y	P (R)	Swelling	N	6 y	NA	IDC	R
63.	F	48	N	P(R)	Swelling	Y	–	N	IDC	R
64.	F	43	N	P (L)	Painless swelling	Y	–	N	IDC	R
65.	F	NA	Y	P (NA)	NA	N	11 mo	NA	IDC	R
66.	F	71	Y	P (L)	Painful mass	N	26 y	N	IDC	R
67.	F	57	Y	To (L)	Painful mass	N	7 mo	Lung	MPT	L
68.	F	60	NA	G (Mand, L, Post)	Hard elastic mass	NA	NA	Lung, spine	MPT	L
69.	F	48	Y	P (R)	Swelling	N	11 y	N	IDC	L
70.	F	36	Y	P (R)	FNP	N	11 mo	MM	IDC	R
71.	F	60	Y	P (R)	Swelling	N	9 mo	N	IDC	R
72.	F	54	Y	P (R)	Swelling, FNP	N	11 mo	MM	IDC	R
73.	F	54	N	P (L)	Swelling	Y	–	Bone	IDC	L
74.	F	60	Y	To (L)	Swelling	N	2 y	N	IDC	R
75.	M	88	Y	RM (L)	Pedunculated mass	N	12 y	N	IDC	NA
76.	F	59	Y	P (L)	Swelling	N	8 y	N	IDC	R
77.	F	55	Y	P (L)	Swelling, Bell's palsy	N	11 mo	N	IDC	L
78.	F	60	Y	P (R)	Lump	N	20 y	Vertebrae	IDC	L
79.	F	39	Y	P (L)	Lump	N	3.5 y	Bone	IDC	L
80.	F	50	Y	P (BL)	Swelling	N	9 y	MM	IDC	L
81.	F	49	Y	T (Base)	Exophytic, ulcerative mass	N	I y	N	IDC	R
82.	F	43	Y	G (Mand, R, Ant)	Ulcerative mass	N	2 y	N	IDC	R
83.	F	52	Y	P (L)	Lump	N	1 Wk.	N	IDC	R
84.	F	68	Y	G (Mand, R, Ant)	Ulcerated, exophytic, erythematous mass	N	8 y	Brain	IDC	NA
85.	F	29	Y	BM (L)	Swelling	N	2 mo	N	IDC	L
86.	F	59	Y	P (L)	Swelling	N	6 y	MM	IDC	L
87.	F	77	Y	G (Mand, R, Post)	Round sessile nodule	N	5 y	N	IDC	L
88.	F	44	Y	Multiple sites	Exophytic, erythematous	N	2 y	MM	TN	L
89.	F	42	Y	BM (R) extending to Palate (L)	Fungating mass	N	6 mo	Skull, neck	IDC	L
90.	F	68	Y	G (Max, R, Post)	Pain, erythematous swelling	N	6 mo	MM	IDC	NA
91.	F	66	Y	BM (R)	Hard mobile mass	N	1 mo	MM	IDC	L
92.	F	37	Y	G (Max, L, Ant)	Erythematous mass	N	9 mo	MM	IDC	L
93.	F	50	Y	SLG	Swelling	N	3 y	MM	IDC	L
94.	F	40	Y	SMG (L)	Swelling	N	10 y	N	ILC	R
95.	F	69	N	P (L)	Swelling	Y	–	Bone	ILC	R
96.	F	50	N	P (L)	Swelling	Y	–	Axillary LN	IDC	L

Abbreviations: Ant, anterior; AS, angiosarcoma; BC, breast cancer; BL, bilateral; BM, buccal mucosa; BP, Bell's palsy; CP, cystosarcoma phyllodes; F, female; FNP, facial nerve palsy; FOM, floor of mouth; G, gingiva; HS, hemangiosarcoma; IDC, invasive ductal carcinoma; ILC, invasive lobular carcinoma; L, left; LN, lymph node; M, male; MM, multiple metastasis; Mand, mandible; Max, maxilla; MPT, malignant phyllodes tumor; NA, not available; P, parotid; Post, posterior; PS, phyllode sarcoma; R, right; RM, retromolar; SLG, sublingual gland; SM, sarcomatous; SMG, submandibular gland; SNA, site not available; T, tongue; TN, triple negative; To, tonsil; UD, undifferentiated; Y, yes.

**Table 3:** Immunoprofile of patients with breast cancer metastasizing to oral soft tissues (September 1967–September 2023)

Patient no.	Immunoreactivity of tumor cells
1.	NA
2.	NA
3.	NA
4.	NA
5.	NA
6.	NA
7.	NA
8.	NA
9.	NA
10.	NA
11.	NA
12.	NA
13.	NA
14.	NA
15.	NA
16.	NA
17.	NA
18.	NA
19.	NA
20.	NA
21.	NA
22.	NA
23.	NA
24.	+ve (ER, PR)
25.	NA
26.	+ve (ER, PR, EMA, GCDFP-15) −ve (S-100)
27.	NA
28.	NA
29.	NA
30.	NA
31.	NA
32.	+ve (CK-7)
33.	NA
34.	NA
35.	NA
36.	NA
37.	NA
38.	NA
39.	+ve (CK-7, CK-14) −ve (ER, PR, HER2)
40.	NA
41.	NA
42.	NA
43.	−ve (ER, PR)
44.	+ve (ER, PR) −ve HER2)
45.	+ve (CD-31, CD-34) −ve (HMG-45, S-100)
46.	+ve (ER) −ve (C-erb2)
47.	NA
48.	NA
49.	NA
50.	+ve (ER, PR)
51.	+ve (ER, PR, ki67)
52.	NA
53.	NA
54.	NA
55.	+ve (CK-AE1/AE3, CAM5)
56.	−ve (ER, PR, ki67, C-erb2)
57.	NA
58.	+ve (ER, PR, ERB2, Ki67)
59.	NA
60.	+ve (ER, PR, ERB2, Ki67, CD56, neuron-specific enolase, synaptophysin chromogranin −ve (C-kit, GCDFP-15)
61.	+ve (ER, PR) −ve (HER2/neu-BRCA1, BRCA2)
62.	+ve (ER)
63.	+ve (ER, PR) −ve (HER2/neu)
64.	NA
65.	NA
66.	+ve (CK-4)
67.	NA
68.	+ve (S-100, p63, Vimentin)
69.	NA
70.	NA
71.	NA
72.	+ve (ER, PR) −ve (HER2/neu)
73.	−ve (Cebr2)
74.	+ve (CK-7, S-100, PR, Mammaglobin) −ve (ER)
75.	+ve (ER, PR)
76.	+ve (ER, PR, HER2neu)
77.	−ve (ER, PR, HER2neu)
78.	+ve (ER, PR, CK-7) −ve (Her-2, GCFP-15)
79.	+ve (ER) −ve (PR, HER2, Ki67)
80.	NA
81.	+ve (CK-14, CK-7) −ve (ER, PR, HER2)
82.	NA
83.	+ve (ER, PR) −ve (HER2)
84.	+ve (HER2) −ve (ER, PR)
85.	+ve (ER, PR) −ve (HER2)
86.	+ve (HER2) −ve (ER, PR)
87.	NA
88.	+ve (CK-AE1/3, CAM5, 2, CK7, S100) −ve (GCDFP15, CD115, Calponin, SMA, ER, PR, HER2)
89.	−ve (ER, PR, HER2)
90.	NA
91.	+ve (ER, PR) −ve (HER2)
92.	+ve (Pan CK)
93.	+ve (CK-7) −ve (ER, PR, HER2)
94.	NA
95.	+ve (ER, PR, CK-7, Pan CK, Mammaglobin) −ve (GCDFP-15, CD20, CD3, CD5)
96.	NA

Abbreviations: BRCA, breast cancer antigen; CD, cluster differentiation; CK, cytokeratin; EMA, epithelial membrane antigen; ER, estrogen receptor; ERB2: receptor tyrosine kinase-2; GCDFP, gross cystic disease fluid protein; HER2, human epidermal growth factor receptor 2; HMG, human menopausal gonadotropin; NA, not available; PR, progesterone receptor; SMA, smooth muscle antigen.

**Table 4:** Data describing treatment and prognosis of patients with breast cancer metastasizing to oral soft tissues (September 1967–September 2023)

Patient no.	Treatment given	Prognosis	Survival time from diagnosis of metastasis to death in months
1.	NA	NA	NA
2.	NA	NA	NA
3.	NA	NA	NA
4.	NA	NA	NA
5.	NA	NA	NA
6.	Radiotherapy	NA	NA
7.	Combined	NA	NA
8.	Combined	NA	NA
9.	NA	NA	NA
10.	NA	NA	NA
11.	NA	NA	NA
12.	NA	NA	NA
13.	Radiotherapy	NA	NA
14.	Combined	NA	NA
15.	NA	NA	NA
16.	NA	NA	NA
17.	Palliative	NA	NA
18.	NA	NA	NA
19.	Combined	D	2
20.	NA	D	15
21.	NA	Fav	–
22.	NA	NA	NA
23.	NA	NA	NA
24.	Hormonal	NA	NA
25.	Palliative	NA	NA
26.	NA	NA	NA
27.	NA	NA	NA
28.	NA	NA	NA
29.	Palliative	D	5
30.	Combined	D	5
31.	Palliative	Fav	–
32.	Palliative Radiotherapy	NA	NA
33.	Extraction	UFU	–
34.	Radiotherapy	NA	NA
35.	NA	NA	NA
36.	Combined	NA	NA
37.	Combined	NA	NA
38.	Combined	Fav	–
39.	Combined	D	12
40.	Combined	D	1
41.	Refused by patient	NA	–
42.	Combined	NA	–
43.	NA	NA	NA
44.	Hormonal	UFU	–
45.	Combined	UFU	–
46.	Combined	LFU	–
47.	Surgery	Fav	–
48.	Palliative	NA	NA
49.	Palliative	D	5
50.	NA	NA	NA
51.	Hormonal	TGO	–
52.	Palliative	NA	NA
53.	Supportive	D	NA
54.	Palliative	D	8
55.	Combined	D	3
56.	Chemotherapy	D	2.5
57.	Combined	Fav	–
58.	Supportive	UFU	–
59.	Hormonal	NA	NA
60.	Combined	NA	NA
61.	Combined	Fav	–
62.	Combined	NA	NA
63.	Combined	NA	NA
64.	Combined	NA	NA
65.	Palliative	NA	NA
66.	Hormonal	Fav	–
67.	Refused by patient	D	1
68.	Death before treatment	–	–
69.	Combined	Fav	–
70.	Hormonal	Fav	–
71.	Combined	NA	NA
72.	Chemotherapy	Fav	–
73.	Combined	Fav	–
74.	Combined	Fav	–
75.	NA	NA	NA
76.	Combined	Fav	–
77.	Combined	NA	NA
78.	Palliative	Fav	–
79.	Combined	NA	NA
80.	Combined	D	12
81.	Combined	NA	–
82.	Combined	Fav	–
83.	Refused by patient	LFU	–
84.	Chemotherapy	D	–
85.	Palliative	NA	NA
86.	Chemotherapy	D	2
87.	Referred to Oncologist	NA	–
88.	Combined	D	5
89.	Palliative	D	1
90.	Death before treatment	–	–
91.	Hormonal	Fav	–
92.	Chemotherapy	D	14
93.	Combined	UFU	–
94.	NA	NA	NA
95.	Combined	Fav	–
96.	Combined	Fav	–

Abbreviations: D, death; Fav, favorable; LFU, lost to follow-up; NA, not available; TGO, treatment going on; UFU, under follow-up.

## Results

Our research strategy revealed a total of 88 relevant papers 7 8 9 10 11 12 13 14 15 16 17 18 19 20 21 22 23 24 25 26 27 28 29 30 31 32 33 34 35 36 37 38 39 40 41 42 43 44 45 46 47 48 49 50 51 52 53 54 55 56 57 58 59 60 61 62 63 64 65 66 67 68 69 70 71 72 73 74 75 76 77 78 79 80 81 82 83 84 85 86 87 88 89 90 91 92 93 94 from September 1967 to September 2023. The results were expressed in descriptive statistics ( Table 4 ). A total of 96 patients were included with 83 females and 1 male with a female to male ratio of 83:1. In 12 cases, no specific gender was documented. The maximum number of cases were from the United Kingdom (n-16) followed by India (n-15), United States (n-7), Israel (n-6), Italy and Japan (n-5). The patients' average age was 54.4 years (range: 24–89). The mean age was 53.8 years in females, and the age of male patients was 88 years. A total of 68 of the 96 patients (70.8%) had a previous history of BC, whereas 17 (17.7%) had none. The most predominant site of OSTM was salivary glands (56.3%) > gingiva (16.7%) > tonsils (7.3%) tongue (6.3%) > buccal mucosa (5.2%). Swelling/lump/mass were the most predominant symptoms (62.5%) followed by ulcerative, pedunculated and nodular lesions. OST was the initial site of metastasis in 17.7% of individuals, the only site of metastasis in 32.3% of cases, whereas 33.3% of cases involved other distant sites too. The most common type of BC diagnosed was invasive ductal carcinoma (IDC) followed by invasive lobular carcinoma (ILC). Major therapeutic aids included were combined therapies (35.4%) and palliative therapy (12.5%). A total of 20.8% of patients died with a mean survival rate of 1 to 15 months ( Table 5 ).

**Table 5:** Summary of results documented from literature research describing the characteristics of patients with breast cancer metastasizing to oral soft tissues (September 1967–September 2023)

Feature	Number/percentage
Total number of papers published	88 • Case reports-82 • Retrospective analysis-3 • Original research-2 • Case series-1
Total number of patients	96
Worldwide distribution of cases	• UK-16 (16.7%) • India-15 (15.6%) • United States-7 (7.3%) • Israel-6 (6.2%) • Italy = Japan-5 (5.2%) • Brazil = Turkey-4 (4.2%) • Germany = Poland = Spain = Tunisia-3 (3.1%) • Belgium = Iran = Nigeria-2 (2.1%) • Africa = Australia = Canada = Cyprus = China = Durham = Finland = Greece = Kuwait = Indonesia = Ireland = Lebanon = Portugal = Senegal = South Korea = West indies-1 (1%)
Gender	• F-83 (86.5%) • M-1 (1%) • NA-12 (12.5%)
Average age of patients (range)	• Total-54.4 (24–89) • Females-53.8 (24–89) • Males-88
Previous history of BC	• Y-68 (70.8%) • N-17 (17.7%) • NA-11 (11.5%)
Site of oral metastasis	• Salivary Glands-54 (56.3%) - P-46 (R-18, L-23, BL-2 SNA-3 - SMG-7 (L-2, R-1 SNA-4 - SLG-1 • Gingiva-16 (16.7%) (Max-7, Mand-5, SNA-3, BL-1) - Max (Ant-4, Post-,1 SNA-2), (R-2, L-2,, SNA-3) - Mand (Ant-2, Post-3), (R-3, L-2) • To-7 (7.3%), L-4 R-2, BL-1 • T-6 (6.3%) • BM-5 (5.2%). L-2, R-3 • RM-MS = 2 (2.1%) • FOM = vestibule = submental region-1(1%) • NA-1 (1%)
Clinical features	• Swelling/mass/lump-60 (62.5%) • Ulcerative lesions-8 (8.4%) • FNP-7 (7.3%) • Exophytic growth-5 (5.2%) • Pedunculated mass-4 (4.2%) • Nodular-2 (2.1%) • Epulis-like growth = BP = hypoesthesia-1 (1%)
Oral soft tissues as the initial site of metastasis	• Y-17 (17.7%) • N-68 (70.6%) • NA-11 (11.5%)
Any other site of metastasis	• Y-32 (33.3%) • N-31 (32.3%) • NA-33 (34.3%)
Average time of detection of metastasis after diagnosis of BC	• 1 wk to 26 y
Type of BC	• IDC-68 (70.8%) • NA-8 (8.3%) • ILC-6 (6.2%) • MPT-4 (4.2%) • AS = CP = TN-2 (2.1%) • HS = UD = PS = SM-1 (1%)
Treatment aids	• Combined-34 (35.4%) • Palliative-12 (12.5%) • Hormonal-7 (7.3%) • Chemotherapy-5. (5.2%) • Radiotherapy-3 (3.1%) • Supportive-2 (2.1%) • Palliative radiotherapy = surgery = extraction-1 (1%) • RBP-3 (3.1%) • DBT-2 (2.1%) • RTO-1(1%) • NA-24 (25%)
Prognosis	• D-20 (20.8%) • Fav-18 (18.7%) • UFU-5 (5.2%) • LFU-2 (2.1%) • TGO-1 (1%) • NA-50 (52.1%)
Average time of death from diagnosis of oral metastasis	• 1–15 mo

Abbreviations: Ant, anterior; AD, adenocarcinoma; AS, angiosarcoma; BC, breast cancer; BL, bilateral; BM, buccal mucosa; BP, Bell's palsy; CP, cystosarcoma phyllodes; D, death; DBT, death before treatment; F, female; Fav, favorable; FNP, facial nerve palsy; FOM, floor of mouth; G, gingiva; HS, hemangiosarcoma; IDC, invasive ductal carcinoma; ILC, invasive lobular carcinoma; L, left; LFU, lost to follow-up; M, male; MM, multiple metastasis; Mand, mandible; Max, maxilla; MPT, malignant phyllodes tumor; NA, not available; P, parotid; Post, posterior; PS, phyllode sarcoma; R, right; RBP, refused by patient; RTO, referred to oncologist; RM, retromolar; SLG, sublingual gland; SM, sarcomatous; SMG, submandibular gland; SNA, site not available; T, tongue; TN, triple negative; To, tonsil; TGO, treatment going on; UD, undifferentiated; UFU, under follow-up; Y, yes.

## Discussion

BC is the first and second leading cause of cancer-related death in developing and developed countries, respectively. In the past few years, the cases of BC have rapidly increased in developed countries, mostly Australia, Western Europe, and Northern America. 2 In the current research, the maximum number of cases were from the United Kingdom (16.7%) followed by India (15.6%), United States (7.3%), Israel (6.2%), Italy and Japan (5.2%). Other regions involved a few cases ( Table 4 ).

BC occurs predominantly during the fifth to sixth decade. 1 In the current study, the age ranged between second and eighth decade. Multiple underlying causes favor the development of BC that include obesity, hormonal and reproductive risk factors, alcohol, drug usage, malnutrition, genetic mutations, etc. 2 In the current research, there were not many associated risk factors, only a few patients had a history of obesity, hypertension, and a family history of BC.

Distant metastasis is the most common cause of death in BC patients. Distant spread of BC most often occurs in the lungs, bones, liver, and brain. The oral cavity is the rarest site. If this occurs, the JB is more affected than OST. 3 Pathogenic mechanisms of metastasis to the OST are not completely recognized. The route of secondary metastasis may be either hematogenous, lymphatic, or direct invasion. BC spreads to the OST predominantly following the hematogenous route. One of the proposed pathways is via Batson's valve plexus system. 51

In the current research, we could document 96 cases of BC metastasizing to OST to date. The first case was reported in 1967. 7 The most common OST involved were salivary glands followed by gingiva, tonsils, tongue, and buccal mucosa. Parotid was the most common gland affected (n-46) followed by the submandibular gland (n-7). Only one case involved the sublingual gland.

Chronically, the inflamed mucosa of the gingiva, particularly the attached gingiva, contains a dense capillary network that can trap malignant cells and promote metastases. 4 In the current research, Studies conclude that gingival metastasis mostly occurs in the mandibular area rather than the maxillary with predominancy of posterior side involvement. In the current research, however, there was maxillary predilection (n-7). The anterior region was mostly affected in the maxilla, whereas there was more involvement of the posterior side in the mandible. In the maxilla, both the right and left sides were affected equally, whereas in the mandible, the right side predominated more than the left. Tonsils are the rarest site of metastasis. According to research, only 0.8% of malignant palatine tonsillar tumors were from an extra-tonsillar source. 3 Lymphatic spread to tonsils is rare due to the lack of afferent lymphatic capillaries except for retrograde spread via cervical lymph nodes or direct spread, the metastatic pathway is unclear. In the current literature, only seven cases of palatine tonsillar metastasis from BC have been observed. The tongue is a highly circulatory organ, which creates ideal conditions for the spread of cancer. The posterolateral and dorsal parts are more often involved in metastasis due to the rich capillary and lymphatic network and immobility. In the current research, 6/96 cases of metastatic BC involved the tongue, maximally affecting the base. Lip, buccal mucosa, the floor of the mouth, retromolar region, palate, and other OST are the rarest sites of metastasis.

Only a few cases involved these regions affected via BC metastasis.

Oral metastatic tumors are of high clinical importance because they may be the only symptom of an undiagnosed underlying malignancy or the first sign of metastasis. 3 4 In our study, 17.7% of cases of OSTM from BC presented as the initial site of metastasis, whereas in 70.6% of cases, metastasis was detected after the mastectomy done for BC, with an average time of 1 week to 26 years. The clinical aspects of BC metastasis in the OST vary according to the anatomical site involved characterized by rapidly growing painful or asymptomatic swellings, lumps or masses, difficulty in chewing, and dysphagia. Facial nerve palsy (FNP) may be a feature of lesions involving salivary glands especially parotid. In our research, seven cases manifested FNP. These metastatic lesions often become difficult to diagnose because their variable appearance bears close resemblance to some benign hyperplastic or reactive oral lesions. In the present research, swelling, lump, and mass were the most predominant clinical features observed. Other lesions appeared as ulcerative, exophytic, pedunculated, nodular, and edematous. A history of primary tumors could help in the detection of secondary metastatic deposits. Before the metastatic spread to the oral cavity, the majority of patients are aware of their primary tumors. However, metastasis to OST via BC is a late indication. In the current research, 70.8% of patients had a previous history of primary BC, whereas 17.7% of patients didn't reveal such a history.

Histopathological examination is required to provide a conclusive diagnosis of the type of metastatic lesion. However, it might be difficult to make an exact diagnosis because of varied histological appearance, particularly when the major focus of the primary site is unknown. Other tools, such as special staining, immunohistochemistry, and electron microscopy, may be necessary in some circumstances to determine the initial tumor's nature. A biopsy is recommended for the histopathological examination to provide a conclusive diagnosis of the type of metastatic lesion. However, it might be difficult to make an exact diagnosis because of varied histological appearance, particularly when the major focus of the primary site is unknown. Histopathologically, BC has been divided into various subgroups. 95 IDC is the most predominant type and has been discovered to be the most prevalent metastasizing to the OST. In the current research, the most prevalent type of metastatic BC was IDC (70.8%) followed by ILC (6.2%). Other types were malignant phyllode tumors, angiosarcoma, hemangiosarcoma, etc. Immunoprofile of the tumor cells in individual patients was also detected, which was variable ( Table 3 ). In many cases, immunohistochemical analysis data were not available.

Imaging techniques such as computerized tomography scans and magnetic resonance imaging can help in the assessment of possible extension or invasion. Positron emission tomography is useful in detecting distant organ metastasis. Although BC entails multiorgan distant metastases, OST might occasionally be the only site of metastasis many times. A total of 32.3% of instances in this study had OST as the only location of BC metastasis, whereas 33.3% had metastasis to other regions as well such as lungs, brain, liver, vertebrae, etc.

The treatment of choice for primary BC ranges from mastectomy to chemotherapy, radiotherapy hormonal therapy, or even palliative treatment. Management for OSTM disease includes a combination of surgical removal of solitary tumors, chemotherapy, radiotherapy, endocrine therapy, and targeted therapy. For single parotid metastasis, parotidectomy (total or superficial) with negative margins (preferably with preservation of facial nerve) and postoperative radiotherapy to obtain local tumor control and to exclude a primary parotid tumor. The most commonly used therapeutic aids in this study were combined therapy (35.4%). Other therapies used were palliative, chemotherapy, radiotherapy, and hormonal. Despite the proposed treatments, patients with metastatic involvement of the OST have poor prognosis, with the 5-year survival rate reported to be 10%. According to the current study, 20.8% of individuals died with an average survival time of 1 to 15 months. A total of 8.7% of patients had a good prognosis with no signs of recurrence. In one patient, treatment is going on. Two cases are under follow-up.

## Limitations of the Current Study

One of the limitations of current research was the small sample size. Most of the studies included were case reports and case series, and in many of the included studies, individual data of patients was not available.

## Conclusions

During the past 56 years (1967–2023), we found only 96 cases of OSTM from BC as the sole primary source. This signifies a rare occurrence of OSTM from BC. The prognosis was poor involving 20.8% deaths with a survival rate of 1 to 15 months. Salivary glands, gingiva, tonsils, tongue, and buccal mucosa were the most prevalent sites to get metastasize. Because of their resemblance to other pathologies and late clinical signs, these lesions go unnoticed the majority of the time. Diagnosis of oral metastatic lesions is a challenging task for the clinicians and pathologists. A thorough examination of the metastatic lesions is required, including a review of the patient's medical history, clinical presentation, and early diagnosis to identify the primary site of metastasis and choose the best course of treatment.
